# Effects of quadric probiotic blends on rumen fermentation, nutrient degradability, and methane emission in sheep: an *in vitro* study

**DOI:** 10.1186/s13568-025-01955-w

**Published:** 2025-10-03

**Authors:** Ali S. A. Saleem, Khaled M. Al-Marakby, Mohamed Y. Elaref, Sabry M. Bassiony, Amera A. Helal, Usama M. Abdel-Monem, Sameh A. Abdelnour

**Affiliations:** 1 Animal Production Department, Faculty of Agriculture, Sohag University, Sohag, Egypt; 2 Animal Production Department, Faculty of Agriculture, Zagazig University, Zagazig, Egypt

**Keywords:** Gas production, In vitro rumen fermentation, Methane emissions, Nutrient degradability, Predictive values, Probiotic combinations

## Abstract

The considerable contribution of ruminant livestock to methane emissions has become a major global concern in recent years. Although dietary approaches for reducing ruminant methane emissions have been explored, the sustainable potential of probiotics to influence rumen function and lower methane production has increasingly attracted research attention. While previous studies have focused on single or dual-strain probiotics, this study is among the first to evaluate the synergistic effects of quadric-strain formulations. Hence, this study aimed to evaluate the impact of multi-strain probiotic blends, each at two distinct concentrations on rumen fermentation, nutrient degradability, and methane emission in sheep using an in vitro gas production technique following a completely randomized design. The basal diet with no probiotic supplements served as a control, while the supplemented bacterial combinations were *Bacillus licheniformis, Lactobacillus acidophilus, L. bulgaricus,* and *Bifidobacterium bifidum* (ABLB; at a ratio of 1:1:1:1) at levels of 2 × 10^9^ (ABLB2) and 4 × 10^9^ (ABLB4) CFU/g of feed, and *Lactobacillus casei, Lactobacillus plantarum, Bacillus subtilis* plus *Bifidobacterium bifidum* (CPSB; at a ratio of 1:1:1:1) at levels of 2 × 10^9^ (CPSB2) and 4 × 10^9^ (CPSB4) CFU/g of feed. Probiotic supplementation significantly improved in vitro dry matter and fiber degradability (IVDMD and IVCFD), with the most effective results observed in ABLB treatments. These blends also reduced methane production and ammonia-N concentrations, while increasing total volatile fatty acids (TVFA), indicating more efficient fermentation. Protozoa counts were notably lower in treated groups, supporting the role of probiotics in mitigating methane via microbial modulation (*P* < 0.01). Probiotic supplementation did not affect the values of pH (*P* > 0.05). Predictive values for metabolizable energy (ME), net energy for lactation (NEL), and organic matter digestibility (OMD) were improved across treatments. These findings highlight the potential of targeted probiotic formulations to enhance rumen efficiency and reduce environmental emissions in ruminant systems.

## Introduction

The rapidly growing global population, coupled with the rising demand for animal protein, poses a significant challenge to food security, particularly in developing countries like Egypt. In Egypt, animal proteins play a fundamental role in human nutrition, and the livestock industry is a vital component of national food security (Rabie 2020; Kamel and El Bilali 2022; Saleem et al. 2024). Recently, in response to the increasing demand for animal proteins and growing public concern over the adverse effects of livestock production, the industry has been under pressure to enhance animal health and production (Hu et al. 2017), minimize environmental harm (Abdelnour et al. 2020; Bashar et al. 2024), and improve the safety of animal-derived products (Kamel and El Bilali 2022).

Feed additives are routinely incorporated into livestock diets to optimize animal performance and maintain optimal health (Abdelnour et al. 2020; Saleem et al. 2025a). For many periods, antibiotics have historically served dual purposes in livestock production, serving both as therapeutic drivers for disease treatment and as growth promoters, thereby enhancing productivity (Robles-Rodríguez et al. 2023). The unruly practice of antibiotics generated antibiotic resistance in animals, with potential implications for resistance in humans. This has prompted a ban, pushing researchers to explore alternative strategies such as probiotics to block infectious disorders while supporting animal growth, health, and welfare (Elghandour et al. 2024). The One Health Joint Plan of Action (OH JPA) against antimicrobial resistance (AMR), launched by the European Union (EU) in 2017, emphasizes the interconnectedness of human and animal health. Consequently, effective AMR management strategies must address both sectors concurrently. Several classifications for the word "probiotic" were implied through the valuable consequences conquered from using various microbial strains in countless host classes (Reuben et al. 2022).

The most widely accepted definition of probiotics comes from the Food and Agricultural Organization (FAO) of the United Nations and the World Health Organization (WHO) (FAO 2016). They define probiotics as “live microorganisms that, when consumed in adequate amounts, confer a health benefit on the host.” As a promising alternative, viable probiotic organisms have shown beneficial effects on animal health, productivity, and performance (Arowolo and He 2018; Ban and Guan 2021). The appropriate utilization of probiotics is establishing them as a safe and environmentally friendly alternative to antibiotics (Devadharshini and Devamugilan 2024). Notably, the synergistic interactions among a blend of different probiotic species, each possessing distinct mechanisms of action, tend to yield superior outcomes compared to the administration of single-strain probiotics (Kulkarni et al. 2022).

Total gas production (TGP) is influenced by microbial protein production, chemical composition, type of diets and the relative amounts of total volatile fatty acids (TVFA) generated during the fermentation process (Krishnamoorthy et al. 1991). Research indicates that butyrate and acetate are key contributors to gas production during this process (Janssen 2010). Moreover, a robust link exists between TVFA production and TGP, particularly when fermented feeds result in elevated acetate levels (Rahman et al. 2013).

Methane emissions from ruminant livestock are a major contributor to anthropogenic greenhouse gases (Saleem et al. 2025a), accounting for approximately 30% of global anthropogenic CH4 emissions (Zhang et al. 2022). Enteric methane production is not only an environmental concern but also results in an energy loss ranging from 2 to 12% of the gross energy intake, thereby negatively impacting animal productivity and feed efficiency (Getabalew et al. 2019).

Various nutritional and microbial strategies have been explored to mitigate methane emissions, with an increasing focus on the use of probiotics due to their natural origin and eco-friendly profile (Saleem et al. 2025a). Probiotics particularly strains of *Lactobacillus*, *Bacillus*, and *Saccharomyces cerevisiae* have shown potential in modulating ruminal fermentation, improving feed conversion efficiency, and altering microbial populations to reduce methanogenesis (Saleem et al. 2025b). Moreover, several studies have reported that dietary probiotic supplementation enhances in vitro TGP and reduces the CH_4_ production (Jeyanathan et al. 2016; Van Lingen et al. 2016; Wang et al. 2016; So et al. 2022; Dhakal et al. 2023). Previous in vitro studies found that probiotic supplementation notably increased the in vitro NH_3_-N concentration (Wang et al. 2016; Abdelbagi et al. 2021; Chang et al. 2021; Marlida et al. 2023), induced a a significant increase in both the TVFA concentration (Sheikh et al. 2017; Abdelbagi et al. 2021; Miguel et al. 2021; Marlidas et al. 2023) during incubation, and the total protozoa numbers (Sızmaz et al. 2020). Additionally, several scientists have shown that supplementation with multi-species probiotic combinations yielded beneficial results on the in vitro dry matter degradability (IVDMD) and in vitro crude fiber degradability (IVCFD) after two days of incubation (Sheikh et al. 2017; So et al. 2022; Marlida et al. 2023). Likewise, a study by Pinloche et al. (2013) documented that the added probiotic increased the total SCFA in the rumen with the cumulative levels of *S. cerevisiae* (10^10^ CFU/g of DM) from 0.5 to 5 g/day. Additionally, a study by Chang et al. (2021) found that the MCP contents were higher with live *B. subtilis natto* (10^9^ CFU) compared to the control after 12 h of in vitro fermentation with dairy rations. *Bacillus subtilis* significantly improved the MCP level by 41.46% relative to the control after one day of in vitro fermentation.

The effectiveness of probiotic bacterial strains is influenced by various critical factors, including the specific species and strains used, the dosage administered, the frequency and timing of supplementation, and overall farm management practices. However, most studies have focused on single-strain probiotic supplementation, and there is limited information on the effects of multi-strain blends, especially in in vitro conditions simulating rumen fermentation. This study aimed to investigate the in vitro effects of two different quadric multi-species probiotic combinations at two inclusion levels (low: 2 × 10^9^ CFU/g feed and high: 4 × 10^9^ CFU/g feed per combination) on nutrient degradability, gas production, methane emission, ruminal fermentation parameters, protozoa count and predicted values.

## Materials and methods

### Location of study and ethical statement

The animal-based experiments for this study were performed at the Animal Nutrition Research Unit, Faculty of Agriculture, Zagazig University, located in Zagazig, Egypt. All experimental procedures and protocols adhered strictly to Directive 2010/63/EU of the European Parliament and of the Council, dated September 22, 2010, which administrates the protection of animals applied for scientific uses.

The investigational methods were authorized by the Sohag University Scientific Research Ethics Committee, the Faculty of Agriculture, Sohag University, Sohag, Egypt (Ethical code: Sohag-IACUC/6/12/1/2024/01). Moreover, all methods, protocols, and animal handling were conducted in accordance with the ARRIVE Guidelines 2.0.

### Experimental design and probiotic combinations

A completely randomized strategy was applied to explore the effects of three quadric combinations of multispecies bacterial strains at two levels/each on rumen fermentation features using the in vitro gas production practice. The basal diet with no probiotic supplements served as a control while the supplemented bacterial combinations were *Lactobacillus bulgaricus, Lactobacillus acidophilus, Bacillus licheniformis* plus *Bifidobacterium bifidum* (ABLB; at a ratio of 1:1:1:1) at levels of 2 × 10^9^ (ABLB2; low) and 4 × 10^9^ (ABLB4; high) CFU/g of feed, and *Lactobacillus casei, Lactobacillus plantarum, Bacillus subtilis* plus *Bifidobacterium bifidum* (CPSB; at a ratio of 1:1:1:1) at levels of 2 × 10^9^ (CPSB2; low) and 4 × 10^9^ (CPSB4; high) CFU/g of feed (Saleem et al. 2025a, b). All the probiotic strain combinations were obtained from Egyptian International Pharmaceutical Industries Co (EIPICO), Tenth of Ramadan City—1st Industrial Zone B1, Egypt, and the preparations were in powder form consisting of the bacteria.

### Diet and proximate chemical examination

The basal diet was formulated based on 50:50 roughage concentrate ratio. The concentrate mixture and berseem hay were finely ground (< 1 mm) and mixed at a percent of 50:50% before used for chemical analysis and in vitro gas production trials. The sample was analyzed for crude protein (CP), dry matter (DM), ash, crude fiber (CF) and ether extract (EE) in fecal and feed samples were assessed conferring to the AOAC (1990). The formulation and proximate chemical composition of the diet is offered in Table 1.

**Table 1:** Design and chemical composition of the basal diet (% on dry matter basis)

Ingredients	%
Yellow corn	35
Berseem hay	50
Limestone	0.6
Mineral and vitamin mixture*	0.15
Wheat barn	6.5
Common salt	0.25
Soybean meal	7.5

Proximate chemical assays (on dry matter basis)			
Substances	Concentrate mixture	Berseem hay	Total mixed diets (calculated)
(g/kg)			
Dry matter	889.3	912.8	901.1
Organic matter	878.4	858	868.2
Crude fibre	95.8	359	227.4
Nitrogen free extract	590.8	331.4	461.1
Crude protein	142	151	146.5
Ether extract	49.8	16.6	33.2
Ash	121.6	142	131.8

*Vitamins and minerals combination consisted of copper 30,000 mg, Iodine 800 mg, Selenium 300 mg, Iron 10,000 mg, MgO 80000 mg, Zinc 100,000 mg, Cobalt 400 mg, Vit. A 10000000 IU, Vit. D_3_ 2,500,000 IU, Vit. E 35000 IU, and CaCO_3_ to 3 kg

### Inoculum donor and preparation

Fresh rumen fluid was collected from four Saidi rams fed a basal diet of 50% concentrate and 50% berseem hay ad libitum for one month before sampling. The rams were fed twice daily at 8:00 AM and 6:00 PM. This feeding regimen ensured consistent microbial activity within the rumen. Prior to morning feeding, rumen fluid was collected from each ram, immediately transferred to pre-warmed containers, and maintained under anaerobic conditions to preserve microbial viability. The rumen samples were collected in the early morning before the morning feeding regimen was applied. The rumen samples were filtered through four layers of cheesecloth and maintained at 39 °C under a CO_2_-saturated atmosphere until used in the in vitro experiments.

### In vitro incubation

The incubation followed a completely randomized design (CRD), comprising four treatments, each with six replicates per run, and the entire procedure was repeated in three independent runs, yielding a total of 30 experimental units per run. Additionally, three blank tubes (without substrate) were included in each run to correct for gas produced by the inoculum itself. No blocking structure was applied.

The buffer solution (MB9 media) contained NaCl (2.8 g/L), CaCl_2_ (0.1 g/L), MgSO_4_.7H_2_O (0.1 g/L), KH_2_PO_4_.H_2_O (2 g/L), and Na_2_HPO_4_ (6 g/L). The pH of the was adjusted to 6.8, and to maintain anaerobic conditions CO_2_ was flushed for 30 min (Onodera and Henderson 1980). A blend of the buffer solution and rumen fluid at a ratio of 2 to 1 (v/v), respectively, was mixed well. Thirty milliliters of the inoculum-buffer mix were dispensed into 50 mL glass tubes containing 200 mg of the ground total mixed diet, quickly locked with gas release rubber plug tailored as designated by Tilley and Terry (1963) with a tri-way valve connected with a calibrated plastic syringe to collect the produced gas. The TGP volume produced during numerous time intervals (3, 6, 12, 24, 36, and 48 h) of incubation was measured using a calibrated syringe. In each run, four blank tubes (without substrate) and six tubes for each treatment were involved. After recording the final gas volume, the CH_4_ emissions were determined using NaOH (10 M) solutions as described by Fievezi et al. (2005). The pH was immediately measured in all replicates using a digital pH meter (model 6010N, Jenco Instruments Inc., San Diego, CA, USA) at the end of each time interval.

### Assessment of partitioning factor, nutrient degradation, ammonia-N, volatile fatty acids concentrations, and protozoa count

Following a 48-h incubation period, the contents of three tubes of each treatment were used to determine the in vitro DM degradability (IVDMD). Thirty mL of neutral detergent solution was added to each tube, mixed well, refluxed for 3 h at 105 °C, filtered through pre-weighed Gooch crucibles, dried at 105 °C for 3 h, and the residual DM weighed as illustrated by Blümmel and Becker (1997). Then, the in vitro crude fiber degradability (IVCFD) was also assessed according to AOAC (1990). For NH_3_-N, TVFA, and protozoa count, the three remaining tubes samples were used. Also, according to Kamra et al. (1991) Protozoa were counted microscopically, while NH_3_-N concentrations were determined through Conway’s method (Conway 1957), and the TVFA concentration was determined according to Warner (1964).

The predictive values for organic matter digestibility (OMD %), partitioning factor (PF), microbial crude protein (MCP; mg/g DM biomass), metabolizable energy (ME; MJ/Kg DM), net energy for lactation (NEL; MJ/Kg DM), and short-chain fatty acids (SCFA; mmol/200 mg DM) were considered using the following equations: (Menke et al. 1979).

$$ \begin{aligned} {\text{OMD }}\left( \% \right) & = 14.88 + \left[ {0.889 \times {\text{GP}}} \right] \\ & \quad + \left( {0.45 \times {\text{CP}}} \right] + \left[ {0.0651 \times {\text{XA}}} \right] \\ \end{aligned} $$

where GP: 24 h net gas production [mL/200 mg), CP: crude protein (%), and XA: ash (%).

PF = total degradable organic matter (mg)/the produced gas (Patra et al. 2017) in 24 h (Blümmel and Becker 1997).

$$ {\text{MCP}} = {\text{DMD}} - [{\text{GP}} \times {2}.{2}] $$

where DMD: DM degradability (mg), GP: gas production (Patra et al. 2017), and 2.2: is a stoichiometric factor that expresses mg of C, H, and O required to produce SCFA gas associated with production of 1 mL of gas.

$$ \begin{aligned} {\text{ME}} & = \left[ {0.157 \times {\text{GP}}} \right] + \left[ {0.0084 \times {\text{CP}}} \right] \\ & \quad + \left[ {0.022 \times {\text{EE}}\left] - \right[0.0081 \times {\text{CA}}} \right] + 1.06 \\ \end{aligned}$$

$$ \begin{aligned} {\text{NEL}} & = \left( {0.115 \times {\text{GP}}} \right) + \left( {0.0054 \times {\text{CP}}} \right) \\ & \quad + \left( {0.014 \times {\text{EE}}} \right) - \left( {0.0054 \times {\text{CA}}} \right) - 0.36 \\ \end{aligned} $$

where GP: 24 h net gas production (mL/200 mg DM), CP: crude protein (%), EE: ether extract and CA: ash (%).

$$ {\text{SCFA}} = \left[ {0.0{222} \times {\text{GP}}} \right] - 0.00{425} $$

where GP: 24 h net gas production (mL/200 mg DM).

### Statistical analysis

Data analysis was achieved using IBM SPSS Software (version 21). The General Linear Model (GLM) technique within SPSS was employed to analyze the gathered data. Analysis of variance (ANOVA) was shown for all investigated parameters. The underlying statistical model is:

$$ Y_{ij} = \mu + \alpha_{i} + e_{ij} $$

where *Y*_*ij*_: an observation, *μ*: the mean, *α*_*i*_: the treatment effect (probiotic combination), and *e*_*ij*_: the standard error. The significant differences among means were analyzed by Duncan’s multiple comparison test (Duncan 1955).

## Results

### Effect on nutrient degradability

**Fig. 1:**
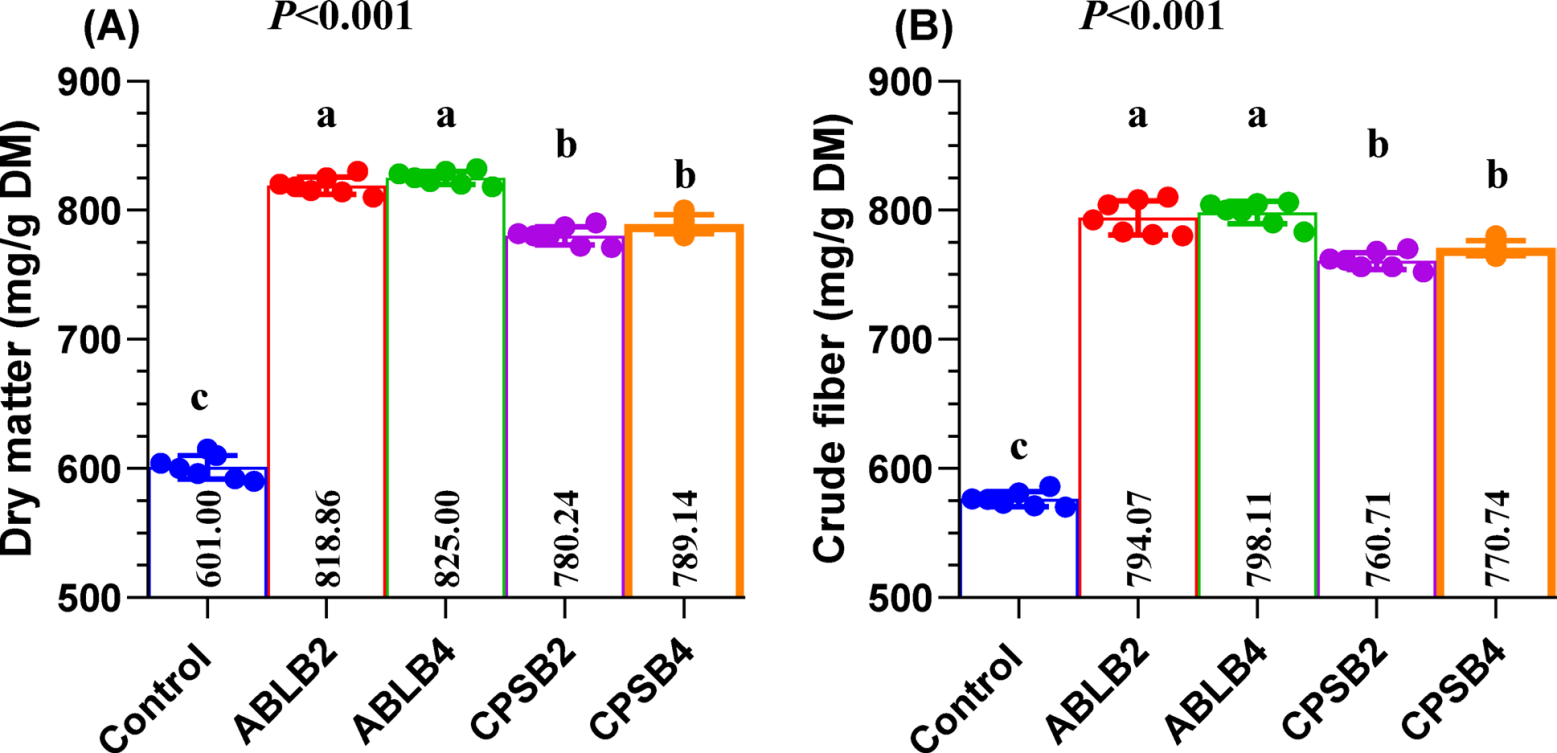
Effect of quadric probiotic combinations on nutrient degradability as dry matter (**A**) and crude fiber (**B**) after 48 h of in vitro incubation. ABLB2 and ABLB4: 2 × 10^9^ CFU/g and 4 × 10^9^ CFU/g of the bacterial formula *Bacillus licheniformis, Lactobacillus bulgaricus, Lactobacillus acidophilus,* and* Bifidobacterium bifidum *CPSB2 and CPSB4: 2×10^9^ CFU/g and 4×10^9^ CFU/g of the bacterial formula *Lactobacillus casei, Bacillus subtilis, Lactobacillus plantarum,* and* Bifidobacterium bifidum. *^a, b, and c^: means within the same column that have different superscripts indicate significant differences (*P* < 0.001).

In vitro incubation over 48 h revealed that all tested probiotic combinations (ABLB and CPSB) significantly increased (*P* < 0.001) IVDMD (Fig. 1A) and crude fiber degradability (IVCFD, Fig. 1B) compared to the control. Specifically, the ABLB combinations, at both low (ABLB2; 2 × 10^9^ CFU/g) and high (ABLB4; 4 × 10^9^ CFU/g) inclusion levels, demonstrated pointedly superior (*P* < 0.001) in the percentage of IVDMD (82.00% and 82.50%, respectively) and IVCFD (79.25% and 79.88%, respectively) than the CPSB combinations (CPSB2: 78.17% and 76.10%; CPSB4: 79.00% and 76.92% for IVDMD and IVCFD, respectively). Within each probiotic combination (ABLB or CPSB), no substantial changes (*P* > 0.05) were observed in IVDMD or IVCFD between the low and high inclusion levels.

### Effects on gas production

Table 2 details the impact of different probiotic bacterial combinations on gas production (mL/g DM) during a 48-h *incubation *in vitro, with measurements taken at 3, 6, 12, 24, 36, and 48 h. Gas production was significantly elevated at all-time points for all probiotic-supplemented groups compared to the control (*P* < 0.001). The ABLB2 and ABLB4 combinations consistently yielded higher gas production values compared with CPSB groups (*P* < 0.001). Notably, the ABLB4 combination exhibited the highest gas production across all measured time points (*P* < 0.01) compared to other groups (expect for ABLB2). Statistical analysis indicated no significant differences in cumulative gas production between the two inclusion levels (2 × 10^9^ CFU/g and 4 × 10^9^ CFU/g) for either probiotic combination at any incubation time. However, a non-significant trend towards increased gas production was observed at the higher inclusion level (4 × 10^9^ CFU/g) compared to low doses (2 × 10^9^ CFU/g) of each type of combination. Overall, probiotic supplementation significantly enhanced gas production relative to the control. The ABLB combination, regardless of inclusion level (ABLB2 and ABLB4), demonstrated comparable total gas production (TGP) and consistently surpassed the CPSB combinations.

**Table 2:** Effect of quadric probiotic combinations on gas production (mL/g DM) at various times (3, 6, 12, 24, 36, and 48 h)

Treatments	Gas production (mL/g DM, at various times, hours)					
	**3**	**6**	**12**	**24**	**36**	**48**
Control	57.10 ± 1.55^c^	93.54 ± 1.59^c^	132.10 ± 1.58^c^	164.36 ± 2.13^c^	192.90 ± 2.76^c^	198.11 ± 2.80^c^
ABLB2	95.85 ± 2.35^a^	168.75 ± 2.70^a^	232.52 ± 2.86^a^	265.61 ± 3.27^a^	278.94 ± 3.67^a^	283.73 ± 4.14^a^
ABLB4	98.77 ± 1.57^a^	170.42 ± 1.36^a^	235.85 ± 1.74^a^	271.65 ± 2.28^a^	280.19 ± 3.19^a^	284.98 ± 2.88^a^
CPSB2	82.31 ± 1.53^b^	150.00 ± 1.44^b^	206.06 ± 1.81^b^	238.73 ± 2.34^b^	252.69 ± 2.47^b^	257.28 ± 2.70^b^
CPSB4	83.98 ± 1.76^b^	155.83 ± 2.45^b^	210.43 ± 4.49^b^	243.94 ± 5.78^b^	255.61 ± 4.79^b^	261.86 ± 4.19^b^
*P* value	0.001	0.001	0.001	0.001	0.001	0.001

ABLB2 and ABLB4: 2 × 10^9^ CFU/g and 4 × 10^9^ CFU/g of the bacterial formula *Bacillus licheniformis, Bifidobacterium bifidum Lactobacillus acidophilus,* and* Lactobacillus bulgaricus.* CPSB2 and CPSB4: 2 × 10^9^ CFU/g and 4 × 10^9^ CFU/g of the bacterial formula *Lactobacillus casei, L. plantarum, Bacillus subtilis,* and* Bifidobacterium bifidum*

^a, b, and c^: means within the same column that have different superscripts indicate significant differences (*P* < 0.05)

### Effects on methane emission

**Fig. 2:**
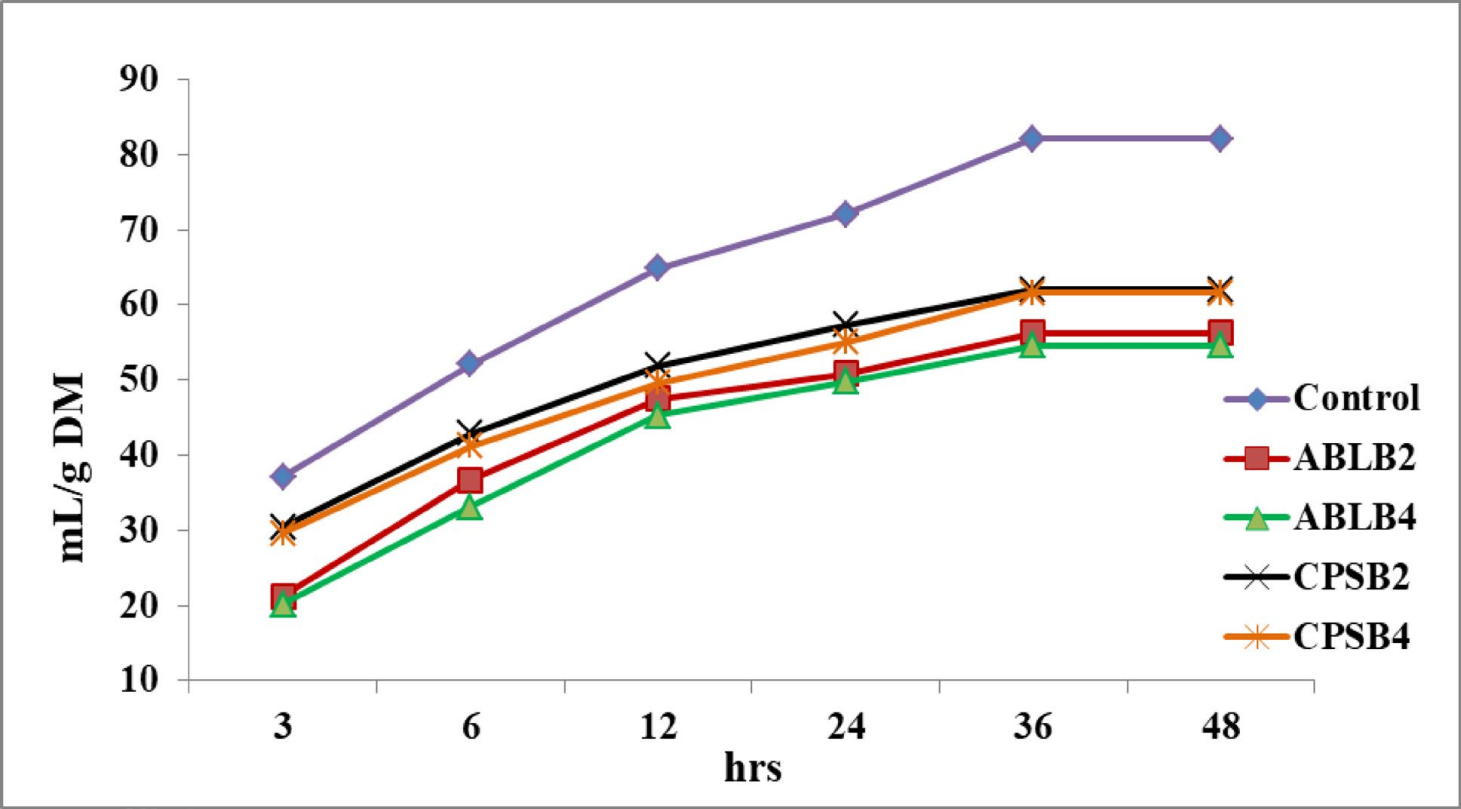
Effect of quadric bacterial combinations on methane emission at various times. ABLB2 and ABLB4: 2 × 10^9^ CFU/g and 4 × 10^9^ CFU/g of the bacterial formula *Bacillus licheniformis, Lactobacillus bulgaricus, Lactobacillus acidophilus,* and* Bifidobacterium bifidum.* CPSB2 and CPSB4: 2 × 10^9^ CFU/g and 4 × 10^9^ CFU/g of the bacterial formula *Lactobacillus casei, Bacillus subtilis, Lactobacillus plantarum,* and* Bifidobacterium bifidum*

Table 3 and Fig. 2 illustrate the effect of four different probiotic bacterial strain combinations on methane (CH_4_) production (mL/g DM) over a 48-h in vitro incubation period, measured at 3, 6, 12, 24, 36, and 48 h. Compared to the control, all probiotic supplements (ABLB2, ABLB4, CPSB2, and CPSB4) significantly reduced (P < 0.001) CH_4_ production throughout the incubation period. The ABLB4 combination consistently exhibited the lowest methane emission values. Significant reductions in CH_4_ production were observed between the ABLB and CPSB treatments at 3 and 6 h of incubation (*P* < 0.001). However, no significant differences in CH_4_ production were observed between the ABLB and CPSB treatments at the remaining incubation times.

**Table 3:** Effect of quadric probiotic combinations on methane emission (mL/g DM) at various times (3, 6, 12, 24, 36, and 48 h)

Treatments	Methane emission (mL/g DM, at various times, hours)					
	3	6	12	24	36	48
Control	37.08 ± 1.50^c^	52.07 ± 2.67^c^	64.81 ± 3.33^b^	72.07 ± 4.27^b^	82.07 ± 4.77^b^	82.07 ± 4.77^b^
ABLB2	21.25 ± 0.91^a^	36.65 ± 1.33^a^	47.52 ± 1.1^a^	50.82 ± 1.24^a^	56.23 ± 1.43^a^	56.23 ± 1.43^a^
ABLB4	20.21 ± 0.68^a^	33.11 ± 0.50^a^	45.23 ± 1.2^a^	49.78 ± 1.76^a^	54.57 ± 1.76^a^	54.57 ± 1.76^a^
CPSB2	30.42 ± 0.77^b^	42.90 ± 0.91^b^	51.89 ± 1.35^a^	57.28 ± 1.3^a^6	62.07 ± 1.47^a^	62.07 ± 1.47^a^
CPSB4	29.58 ± 0.42^b^	41.23 ± 1.36^b^	49.60 ± 2.64^a^	54.98 ± 3.84^a^	61.65 ± 3.62^a^	61.65 ± 3.62^a^
*P* value	0.001	0.001	0.001	0.001	0.001	0.001

ABLB2 and ABLB4: 2 × 10^9^ CFU/g and 4 × 10^9^ CFU/g of the bacterial formula *Bacillus licheniformis, Bifidobacterium bifidum Lactobacillus acidophilus,* and* Lactobacillus bulgaricus.* CPSB2 and CPSB4: 2 × 10^9^ CFU/g and 4 × 10^9^ CFU/g of the bacterial formula *Lactobacillus casei, L. plantarum, Bacillus subtilis,* and* Bifidobacterium bifidum*

^a, b, and c^: means within the same column that have different superscripts indicate significant differences (*P* < 0.05)

### Effects on rumen fermentation parameters and protozoa count

**Fig. 3:**
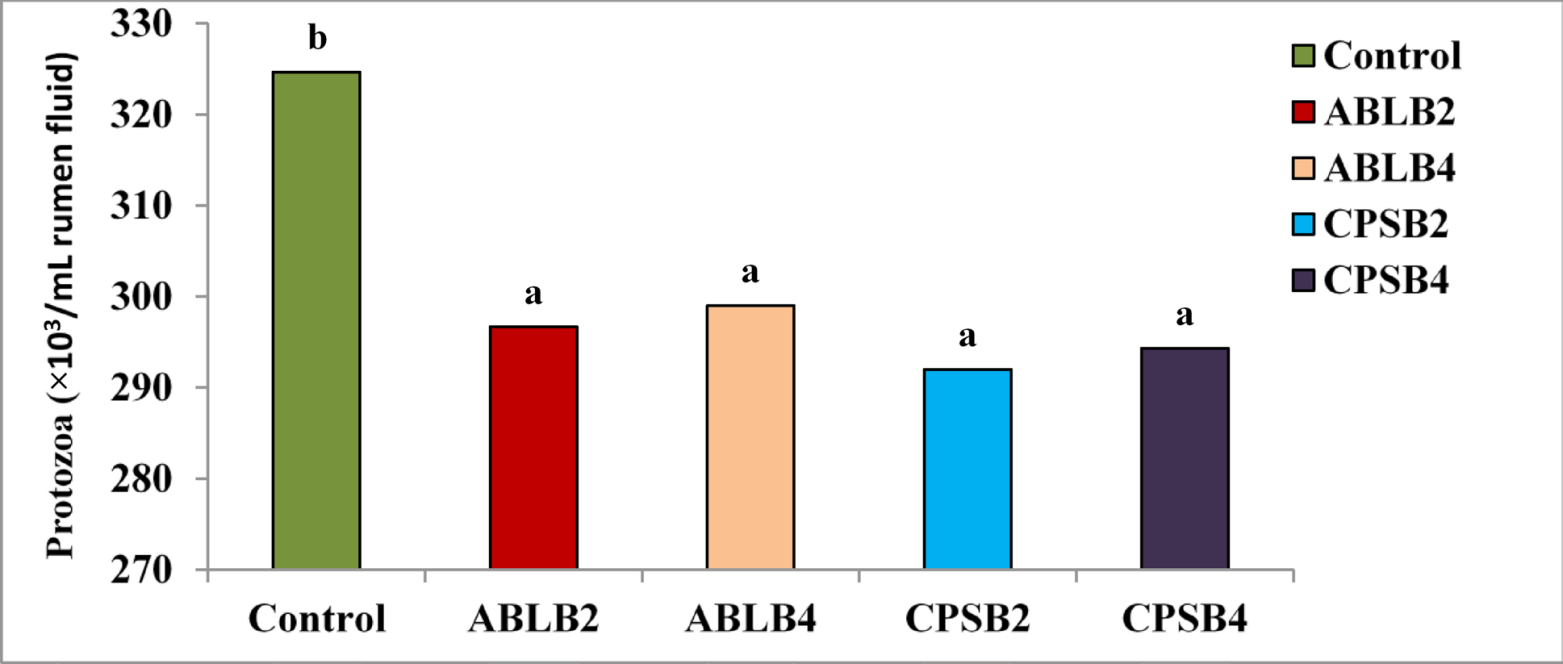
Effect of quadric probiotic combinations on protozoa count. ABLB2 and ABLB4: 2 × 10^9^ CFU/g and 4 × 10^9^ CFU/g of the bacterial formula *Bacillus licheniformis, Bifidobacterium bifidum Lactobacillus acidophilus,* and* Lactobacillus bulgaricus.* CPSB2 and CPSB4: 2 × 10^9^ CFU/g and 4 × 10^9^ CFU/g of the bacterial formula *Lactobacillus casei, L. plantarum, Bacillus subtilis,* and* Bifidobacterium bifidum.* ^a,b^: columns labeled with diverse letters show significant differences (*P* < 0.05)

Table 4 demonstrates that the probiotic combinations had no substantial consequence on ruminal pH values. However, significant differences (*P* = 0.001) were detected in NH_3_-N concentrations, TVFA concentrations, and protozoa count (Fig. 3) between the probiotic treatments (ABLB and CPSB) and the control. Both probiotic combinations, high and low doses together, significantly reduced (*P* = 0.001) NH_3_-N concentrations and protozoa numbers while significantly increasing (*P* = 0.001) TVFA concentrations compared to the control. The ABLB4 combination exhibited the most pronounced improvements in all fermentation parameters, including TVFA concentrations, NH_3_-N concentrations, pH, and protozoa count. Probiotic supplements positively influence the rumen environment by promoting stable fermentation and enhancing its overall development. In ruminant nutrition studies, the assessment of multiple parameters, such as rumen TVFA concentrations, NH_3_-N concentration, and pH, is crucial for evaluating the impact of dietary interventions on the host animal. It is well-established that these parameters are closely interrelated and are significantly influenced by rumen microbial populations, which are directly impacted by both bioactive substances and dietary substrates.

**Table 4:** Effect of quadric bacterial combinations on fermentation parameters

Treatment	Fermentation parameters		
	NH_3_-N (mg/dL)	TVFA (mL eq/L)	pH
Control	38.08 ± 1.12^c^	190.00 ± 4.04^c^	5.90 ± 0.04
ABLB2	25.20 ± 1.26^ab^	220.33 ± 1.45^ab^	5.83 ± 0.02
ABLB4	23.33 ± 1.17^a^	224.67 ± 3.18^a^	5.84 ± 0.03
CPSB2	26.72 ± 1.26^ab^	216.00 ± 2.08^ab^	5.80 ± 0.04
CPSB4	25.67 ± 1.17^ab^	215.00 ± 2.89^ab^	5.81 ± 0.03
*P* value	0.001	0.001	0.162

^a, b, and c^: means within the same column that have different superscripts indicate significant differences (*P* < 0.05). ABLB2 and ABLB4: 2 × 10^9^ CFU/g and 4 × 10^9^ CFU/g of the bacterial formula *Bacillus licheniformis, Bifidobacterium bifidum Lactobacillus acidophilus,* and* Lactobacillus bulgaricus.* CPSB2 and CPSB4: 2 × 10^9^ CFU/g and 4 × 10^9^ CFU/g of the bacterial formula *Lactobacillus casei, L. plantarum, Bacillus subtilis,* and* Bifidobacterium bifidum*

### Predictive values of organic matter digestibility, short chain fatty acids and microbial crude protein production, partitioning factor, and energy for lactation

Table 5 presents the effects of the quadric probiotic combinations on predicted aspects. Compared to the control, all predicted values—OMD, SCFA production, MCP synthesis, ME, NEL—were significantly increased (*P* = 0.001). Conversely, the PF was significantly decreased (*P* = 0.001) with both ABLB and CPSB treatments. The ABLB4 treatment exhibited the highest predicted values (*P* = 0.001) for OMD, SCFA production, ME, and NEL, while displaying the lowest predicted PF values. No significant changes (*P* > 0.05) were detected in MCP synthesis among the probiotic groups. Furthermore, no statistically significant variation was observed between the effects of the two inclusion levels (2 × 10^9^ CFU/g and 4 × 10^9^ CFU/g) within each tested probiotic combination on any of the estimated predictive values.

**Table 5:** Effect of quadric probiotic combinations on predictive values of sheep

Treatments	Predictive values					
	OMD (%)	SCFA (Mmol)	MCP (mg/g DM biomass)	ME (MJ/kg DM)	NEL (MJ/kg DM)	PF (mg TDOM/mL gas)
Control	51.09 ± 0.38^c^	0.73 ± 0.01^c^	525.94 ± 5.93^b^	6.31 ± 0.07^c^	3.48 ± 0.05^c^	1.56 ± 0.01^c^
ABLB2	70.17^±^0.41^a^	1.20 ± 0.01^a^	703.64 ± 5.65^a^	9.68 ± 0.07^a^	5.94 ± 0.05^a^	1.29 ± 0.01^a^
ABLB4	69.10 ± 0.58^a^	1.18 ± 0.02^a^	700.84 ± 13.55^a^	9.49 ± 0.1^a^	5.80 ± 0.07^a^	1.30 ± 0.01^a^
CPSB2	65.25 ± 1.03^b^	1.08 ± 0.02^b^	678.35 ± 14.39^a^	8.81 ± 0.18^b^	5.31 ± 0.13^b^	1.34 ± 0.01^b^
CPSB4	64.32 ± 0.42^b^	1.06 ± 0.01^b^	674.97 ± 14.75^a^	8.65 ± 0.07^b^	5.19 ± 0.05^b^	1.35 ± 0.01^b^
*P*-value	0.001	0.001	0.001	0.001	0.001	0.001

^a, b, and c^: means within the same column that have different superscripts indicate significant differences (*P* < 0.05). Organic matter degradability (OMD), short chain fatty acids (SCFA), partitioning factor (PE), microbial crude protein (MCP), metabolizable energy (ME), and net energy for lactation (NEL). ABLB2 and ABLB4: 2 × 10^9^ CFU/g and 4 × 10^9^ CFU/g of the bacterial formula *Bacillus licheniformis, Bifidobacterium bifidum Lactobacillus acidophilus,* and* Lactobacillus bulgaricus.* CPSB2 and CPSB4: 2 × 10^9^ CFU/g and 4 × 10^9^ CFU/g of the bacterial formula *Lactobacillus casei, L. plantarum, Bacillus subtilis,* and* Bifidobacterium bifidum*

## Discussion

Antimicrobial resistance has emerged as a critical global public health issue. Probiotics are recognized as a sustainable and environmentally friendly strategy for improving animal health and productivity, with the potential to reduce methane emissions. Previous studies have mainly focused on the use of single or dual-strain probiotic blends. However, this study took a novel approach by using blends of four distinct probiotic strains (quadric combinations) at two different inclusion levels. The in vitro evaluation showed that all probiotic combinations significantly increased gas production and reduced methane emissions compared to the control group, with the ABLB treatment showing the most significant effects. Additionally, all tested probiotics significantly decreased NH_3_-N concentrations, pH, and protozoa counts while increasing TVFA concentrations. Predicted values for OMD, SCFA, MCP, ME, and NEL were significantly higher, while PF values were significantly lower across all probiotic treatments. Notably, the ABLB combination consistently produced the most favorable results across all in vitro tested parameters. Importantly, increasing the inclusion level of probiotics did not have a significant impact on any of the evaluated characteristics.

The ABLB combination (*Lactobacillus acidophilus, Lactobacillus bulgaricus, Bacillus licheniformis* and *Bifidobacterium bifidum*) at low (ABLB2) and high (ABLB4) levels resulted in significantly higher IVDMD and IVCFD values compared to CPSB2 and CPSB4 groups (*P* < 0.001). Similarly, several studies have reported that probiotic combinations improve* vitro* nutrient degradability. For instance, authors using *Lactobacillus casei* plus* Lactobacillus lactis* (Baah et al. 2009), a probiotic involving* Lactobacillus acidophilus* (Sheikh et al. 2017), a blend probiotic including* Lactobacillus plantarum* (Marlida et al. 2023), and *L. casei* TH14 all observed this beneficial effect.

As previously reported, strains of *Lactobacillus* and *Bifidobacterium* spp. are commonly employed as dietary probiotic supplements in ruminant diets. The efficacy of these bacterial strains is influenced by several key factors, including the specific bacterial strain(s) selected, the administered dosage, the frequency and timing of supplementation, and overall farm management practices (Puniya et al. 2015). Probiotics exert beneficial stimulatory effects on rumen fermentation, which may contribute to enhanced nutrient digestibility (Ridwan et al. 2018; Saleem et al. 2025b).

*Lactobacillus* species have been shown to significantly correlate with several in vitro fermentation parameters, including IVDMD and IVOMD (Li et al. 2022). *Lactic acid* bacteria (LAB) interact with the rumen microbiota by promoting beneficial fermentative processes and suppressing the growth of pathogenic microbes through the production of antimicrobial molecules such as bacteriocins (Weinberg et al. 2004). It has been proposed that probiotic supplement may boost the ability of ruminal microbes to adapt to the existence of lactic acid or reduce lactic acid accumulation in the rumen by facilitating the conversion of lactic acid to acetic acid (Ghorbani et al. 2002; Nocek et al. 2002). These conditions promote microbial digestion of feed, especially fibrous feedstuffs, and support the activity of cellulolytic bacteria (Jiao et al. 2017). Furthermore, *Bacillus licheniformis* is known to stimulate the growth of cellulolytic bacteria in the rumen, thereby enhancing cellulose digestion (Qiao et al. 2010). Additionally, certain *Bifidobacterium* spp. possess *β*-glycosidase activity, enabling them to cleave the *β*-1-4-glucosidic bonds between glucose molecules in carbohydrates (Pokusaeva et al. 2011). These findings from previous research align with the results of the current study, which may highlight the synergistic effects observed with the ABLB combination.

In the present experiment, the highest values of IVDMD and IVCFD were observed with ABLB2 and ABLB4 which is in agreement with Izuddin et al. (2018), who reported that an increase in TGP suggests a rise in nutrient degradability and the activity of fiber-degrading microbes. Previous research papers have shown that the LAB increased TGP with *Lactobacillus bulgaricus* D1 (Jeyanathan et al. 2016), *Lactobacillus acidophilus* (Chen et al. 2017), *Lactobacillus casei* and *L. plantarum* (Khota et al. 2017). On the other hand, Wang et al. (2016) noted that *B. licheniformis* elevated the TGP (*P* < 0.05). Additionally, Dhakal et al. (2023) confirmed that adding a probiotic formulation involving *B. licheniformis* increased the TGP after 48 h of incubation with grass silage.

Methane emission from livestock is a significant contributor to global climate change. Utilizing green approaches such as probiotics may offer a viable and sustainable strategy for reducing methane releases from livestock. Results indicated that the lowest methane emission value was observed in the ABLB4 group. Several recent research papers, including Philippeau et al. (2017), Astuti et al. (2018), and Abdelbagi et al. (2021) have shown that *L. plantarum* can decrease methane production in vitro. Similarly, Khota et al. (2017) demonstrated that *Lactobacillus casei* significantly (*P* < 0.05) reduced the in vitro methane production. In the same line, Jeyanathan et al. (2016) stated reported a significant effect of *L. bulgaricus* on the in vitro CH_4_ emission. In contrast, Wang et al. (2016) noticed that the *B. licheniformis* reduced the in vitro CH_4_ production (*P* < 0.05). Additionally, Dhakal et al. (2023) found that a combination of *B. licheniformis* and *B. subtilis* (5.9 × 10^7^ CFU/mL) decreased total methane production by 4–6%. However, Janssen (2010) and Van Lingen et al. (2016) have reported that butyrate and acetate are the primary causes of gas production during fermentation.

A study by Bi et al. (2018) observed a positive correlation between the molar proportion of butyrate and the abundance of *Bifidobacterium*. Similarly, Lyons et al. (2018) summarized a strong positive association between the relative abundances of *Bifidobacterium* and the concentrations of acetate and butyrate, leading to increased methane production in cows. These findings are consistent with the results of Astuti et al. (2018), who found that the addition of LAB can promote the growth of LUB, increase propionic acid production, and reduce hydrogen availability for methane synthesis. Differences in bacterial strains or their metabolites can influence their ability to modify rumen fermentation patterns and inhibit specific rumen bacteria responsible for methane production (Doyle et al. 2019).

A study by Philippeau et al. (2017) found that *Lactobacillus spp*. can produce antimicrobial peptides such as bacteriocins that affect CH_4_ emission. The tested probiotics may have a mitigating effect on CH_4_, possibly through altering microbial populations or fermentation pathways. Lower methane emissions are desirable as they indicate more efficient energy utilization by the host animal. Some authors have suggested that *B. licheniformis* and *B. bifidum* can produce propionate and act as a hydrogen sink (Kulkarni et al. 2022; Saleem et al. 2025a). The propionate pathway, shown below, uses hydrogen to reduce pyruvate to propionate. This process diverts hydrogen away from methanogens, depriving them of their main substrate. The current study used an in vitro model to examine the impact of combinations of bacterial strains as probiotics on rumen fermentation. Results of TVFA in the current investigation are in accordance with Baah et al. (2009), who observed a linear increase in TVFA production with increasing inclusion levels of a blended culture of *L. lactis* and* L. casei* compared to the control diet (barley-based diets). Likewise, Paengkoum et al. (2011) concluded that the probiotic combination including* L. acidophilus* plus *S. cerevisiae* (10^10^ CFU/g DM) augmented the average concentration of the TVFA. Additionally, Sheikh et al. (2017) illustrated that the TVFA concentration in rumen fluid was significantly raised compared to the control after 48 h of incubating *L. acidophilus (*6 × 10^9^ CFU/g) and *S. cerevisiae (*2 × 10^10^ CFU/g) with the basal diet (rice straw: concentrate mixture; 50:50%). Also, Miguel et al. (2021) showed that the probiotic consisted of *L. plantarum, *and *S. cerevisiae* (10^10^ CFU/mL) substantially boosted (*P* < 0.05) the TVFA level by 11% linked to the basal diet (rice straw-based rations; 60:40). Moreover, *B. licheniformis* and certain *Lactobacillus* species, are known to produce a range of enzymes like cellulases, xylanases, and pectinases. These enzymes break down complex plant fibers (cellulose, hemicellulose) into simpler sugars that can be fermented more easily (Kulkarni et al. 2022; Saleem et al. 2025a).

At all sampling times, the TGP with the quadric probiotic combinations, ABLB was significantly (*P* = 0.001) better than the other treatment (Table 3). These findings reflect the higher production of TVFA in the ABLB treatments. Krishnamoorthy et al. (1991) demonstrated that TGP is influenced by the composition of feed, microbial protein synthesis, and VFA production during fermentation. Several studies by Janssen (2010) and Van Lingen et al. (2016) have highlighted that acetate and butyrate are the primary sources of gas production during fermentation. Also, Rahman et al. (2013) found that in vitro fermentation of forage resulted in high acetate production. Similarly, Lyons et al. (2018) showed a positive correlation between the relative abundances of *Bifidobacterium* and acetate and butyrate concentrations, indicating elevated TVFA concentrations. Additionally, Qiao et al. (2010) reported that dietary *B. licheniformis* improved TVFA in dairy cows, possibly due to the unique adaptation of *B. licheniformis* in starch hydrolysis.

Regarding NH_3_-N concentrations, both ABLB and CPSB reduced the NH_3_-N concentrations (*P* = 0.001) compared to control group. Limited data are available on the direct measurements of in vitro NH_3_-N concentration affected by the inclusion of bacterial probiotic combinations.

The findings of the current study are consistent with Wang et al. (2016) who detected that *Bacillus subtilis* significantly reduced (*P* < 0.05) NH_3_-N levels compared to the control. Also, Deng et al. (2018) observed that the dietary *B. licheniformis* led to lower ruminal NH_3_-N concentrations compared to control one. Additionally, Sızmaz et al. (2020) reported that a probiotic combination involving* S. cerevisiae, B. subtilis, B. bifidum, L. acidophilus, L. plantarum, L. casei,* and* L. fermentum* resulted in a decrease in in vitro NH_3_-N for 48 h of incubation. Previous studies by Jia et al. (2018) and Chen et al. (2021) discussed how probiotic supplements can enhance the use of NH_3_-N and VFA, indicating better synthesis of microbial protein. Furthermore, a study by Faniyi et al. (2019) suggested that pH value is crucial for maintaining suitable rumen environments, optimal fermentative performance, nutrient digestibility, and overall rumen health.

The ABLB and CPSB quadric probiotic combinations had no significant impact on the pH values compared to the control. Similar results were noticed by Jeyanathan et al. (2016) with *Lactobacillus bulgaricus*, Chen et al. (2017) with *Lactobacillus acidophilus*, Sızmaz et al. (2020) with the probiotic combination including *L. acidophilus, B. subtilis, B. bifidum, L.casei, L. plantarum,* and* L. bulgaricus*, Abdelbagi et al. (2021) and Saleem et al. (2025b). Additionally, Elghandour et al. (2015) clarified that the action of probiotics in the rumen predominantly depends on LAB and LUB. For instance, Nocek et al. (2002) and Yoon and Stern (1995) revealed that LAB produce organic acids that beneficially mitigate acidosis in the rumen by promoting the development of advantageous microbes and LUB. LUB acts by declining lactic acid levels within the rumen later, preserving a stable pH in the rumen (Reuben et al. (2022). As discussed by Kulkarni et al. (2022) noted that multi-strain probiotics have beneficial impacts on ruminant's well-being and productivity by stabilizing the ruminal environment. The quadric probiotic combinations (ABLB and CPSB) significantly reduced the protozoa numbers (*P* = 0.030). Our results are consistent with Sun et al. (2013), who found that Bacillus subtilis natto supplements decreased protozoa count (*P* < 0.01) in rumen fluid. In contrast, Sızmaz et al. (2020) reported that a probiotic formulation containing *S. cerevisiae, B. subtilis, B. bifidum, L. acidophilus, L. casei, L. plantarum,* and* L. bulgaricus* led to a slight but statistically significant (*P* < 0.001) increase in the total numbers of protozoa compared to the control group. Protozoa have been implicated in methane production (Patra et al. 2017), as methanogenic bacteria, particularly those associated with protozoa, consume H2 to produce methane. Therefore, the decrease in protozoa numbers observed in our study may have contributed to the reduction in methane production (Guyader et al. 2014). The improved OMD in existing research may be attributed to the increase in fermentable carbohydrates in diets, which helps in SCFA production in the rumen, especially butyrate and propionate (Siciliano-Jones and Murphy 1989; AlZahal et al. 2017).

The partitioning factor (PF) is an index of the contribution of TDOM between fermentation gases and microbial biomass during fermentation (Blümmel and Becker 1997). The lower PF in this study may be due to a large amount of potentially digestible organic matter remaining undigested but solubilized, erroneously contributing to TDOM, especially when incubations are terminated at times preceding 24 h (Krishnamoorthy and Robinson 2010). Different strains of *Lactobacillus* produce SCFA during microbial fermentation of dietary fiber in the intestinal region (Liu et al. 2021). *Bifidobacterium* is subsp*. lactis* GCL2505 increases SCFA levels in the gut, enhancing host energy expenditure (Horiuchi et al. 2020). Furthermore, a probiotic blend comprising *L. acidophilus, B. bifidum, L. plantarum, and L. bulgaricus* led to an insignificant increase in total SCFA concentrations (*P* > 0.05) compared to the control (Sızmaz et al. 2020).

Propionate is a gluconeogenic SCFA in ruminants. In this sense, Liu et al. (2022) discussed that SCFA provides greater than 70% of the metabolizable energy supply in ruminants as a major energy source. The propionate fermentation pathway differs from those that produce acetate and butyrate because it does not release hydrogen. As a result, enhancing propionate production could help lower CH_4_ emissions from the rumen. Additionally, Moran (2005) explained that the concentrations of the primary SCFAs in the rumen are heavily influenced by diet. Two studies Van Soest (1994) and Moran (2005) elucidated that diets rich in fibrous components tend to result in a higher acetate-to-propionate ratio, which generates more CH_4_ production and consequently leads to energy loss in ruminants. In the current trial the tested diet consisted of berseem: concentrates; 50:50%. The ABLB combination increased the TGP (Table 3), while decreased the CH_4_ release which may indicate a inferior proportion of acetate-to-propionate. The same context, Liu et al. (2022) supposed that probiotics may improve ruminal SCFA creation, possible enhancing the net energy utilization. A greater acetate-to-propionate ratio affects both milk fat content and overall animal recital. Contrariwise, a higher propionate-to-acetate ratio (or a lower acetate-to-propionate ratio) improve growth performance and nutrient utilization efficiency. According to Lu et al. (2019), the SCFA are the most prevalent microbial metabolites in rumen. Ruminal SCFA promotes the transfer of blood urea into the rumen. When the diet includes 28% non-fiber carbohydrates, the increased transport of urea into the rumen and improved nitrogen recycling are driven by greater microbial diversity in the rumen, as indicated by higher SCFA concentrations.

Due to limited institutional resources, our study faced certain constraints. To further validate our promising findings, in vivo trials are essential to confirm the beneficial effects of the probiotic combinations in sheep diets. Additionally, incorporating more advanced molecular analyses, such as 16S rRNA sequencing of the microbial community in the sheep rumen, would greatly enhance our understanding of probiotic actions at a genetic level and shed light on their impact on the microbial ecosystem.

## Conclusion

This study provides evidence that incorporating quadric multi-species probiotic combinations (*lactobacillus acidophilus, Lactobacillus bulgaricus, Bacillus licheniformis* + *Bifidobacterium bifidum*) at 2 × 10^9^ CFU/g into sheep diets can significantly modulate rumen fermentation, enhance nutrient utilization, mitigate methane emissions, and foster a more favorable rumen ecosystem. Notably, the ABLB combination consistently exhibited superior performance compared to CPSB across key parameters, including nutrient degradability, methane reduction, and predicted values for OMD, SCFA production, and MCP synthesis. These findings support the integration of multi-strain probiotics in sheep nutrition to improve feed utilization and environmental sustainability, offering a viable alternative to antibiotics. The observed synergistic effects within these multi-species blends underscore their capacity to substantially decrease the environmental footprint of livestock production while concurrently improving animal health and productivity. To confirm these results, in vivo validations are required. These should be accompanied by omics tools to provide insight into how these additives modulate the rumen microbiota.

## Data Availability

Upon reasonable request, the corresponding author will provide the data from this study.
